# Determination of Maximal Oxygen Uptake Using the Bruce or a Novel Athlete-Led Protocol in a Mixed Population

**DOI:** 10.2478/v10078-012-0010-z

**Published:** 2012-04-03

**Authors:** Michael J. Hamlin, Nick. Draper, Gavin Blackwell, Jeremy P. Shearman, Nicholas E. Kimber

**Affiliations:** 1 Department of Social Science, Parks, Recreation, Tourism and Sport, Lincoln University, Christchurch, New Zealand.; 2 School of Sciences and Physical Education, University of Canterbury, Christchurch, New Zealand; 3 School of Applied Sciences and Allied Health, Christchurch Polytechnic Institute of Technology, Christchurch, New Zealand.

**Keywords:** V̇O_2max_, treadmill, running time, aerobic performance

## Abstract

Treadmill tests for maximal oxygen uptake (V̇O_2max_) have traditionally used set speed and incline increments regardless of participants training or exercise background. The aim of this study was to determine the validity of a novel athlete-led protocol for determining maximal aerobic fitness in adults. Twenty-nine participants (21 male, 8 female, age 29.8 ± 9.5 y, BMI 24.4 ± 3.1, mean ± SD) from a variety of exercise backgrounds were asked to complete two maximal treadmill running tests (using the standard Bruce or a novel athlete-led protocol [ALP]) to volitional failure in a counter-balanced randomised cross-over trial one week apart. We found no substantial difference in maximal oxygen uptake (47.0 ± 9.1 and 46.8 ± 10.7 ml·kg^−1^·min^−1^, mean ± SD for the ALP and Bruce protocols respectively), evidenced by the Spearman correlation coefficient of 0.93 (90% confidence limits, 0.88-0.96). However, compared to the Bruce protocol, participants completing the ALP protocol attained a substantially higher maximal heart rate (ALP = 182.8 ± 10.5, Bruce = 179.7 ± 8.7 beats·min^−1^). Additionally, using the Bruce protocol took a longer period of time (23.2 ± 17.0 s) compared to the ALP protocol. It seems that using either treadmill protocol will give you similar maximal oxygen uptake results. We suggest the ALP protocol which is simpler, quicker and probably better at achieving maximal heart rates is a useful alternative to the traditional Bruce protocol.

## Introduction

Maximal aerobic power is commonly accepted as the best measure of the cardiovascular systems’ functional limits (Rowell, 1974) and has been shown to predict mortality from all causes in healthy (Blair et al., 1989; 1996) and unhealthy individuals (Myers et al., 2002). Accurate and reliable measurement of maximal aerobic fitness is therefore essential not only for healthy but also unhealthy individuals if the results are to be used in any health screening process.

Many measures of aerobic fitness traditionally utilize sub-maximal exercise tests which use algorithms to predict maximal aerobic fitness (Grant et al., 1995). A more complicated but also more accurate measure of maximal aerobic fitness is to directly measure individual’s oxygen uptake during a progressive increase in exercise intensity. However, protocols for such testing are inconsistent, complicated, and can produce quite dissimilar maximal aerobic fitness results (Froelicher et al., 1974; Pollock et al., 1976; Yoon et al., 2007).

The maximal oxygen uptake (V̇O_2max_) test was first standardized by Taylor and colleagues in 1955 (Taylor et al., 1955) when these researchers used a discontinuous protocol to progressively increase work rate over a number of days. Taylor et al. (1955) had participants run for 3 minutes on a treadmill at a speed of 7 mph. Over a number of consecutive days the treadmill grade was gradually increased by 2.5% until the increment in oxygen uptake was less than 150 ml per minute (the so called ‘plateau’ effect) reflecting V̇O_2max_. Subsequently Shephard et al. (1968) found that a continuous test (using 2-minute increments of work rate beginning at 90–100% predicted V̇O_2max_) was able to produce similar V̇O_2max_ values (Shephard et al., 1968). Since the description of these initial protocols a plethora of procedures have been used in measuring V̇O_2max_, many with regimented and complicated steps or instructions (Astrand and Rodahl, 1970; Balke and Ware, 1959; Ellestad et al., 1969; McDonough and Bruce, 1969). The publication of guidelines for exercise testing by the American College of Sports Medicine in 1975 has in some ways helped to standardise testing procedures (American College of Sports Medicine, 1975), however complicated V̇O_2max_ testing protocols continued to develop (Buchfuhrer et al., 1983; Davis et al., 1982; Fairshter et al., 1983; Kang et al., 2001). Therefore, the aim of this study was to examine and compare V̇O_2max_ results from the popularly-used Bruce protocol (Bruce, 1971) against a simplified and less restrictive novel athlete-led protocol with the same subject population.

## Material and Methods

### Participants

Twenty-nine participants (21 male, 8 female) from a variety of exercise backgrounds volunteered to participate in the present study. Participant characteristics are presented in Table 1. Twenty-two of the participants had no prior experience of completing maximal running tests to exhaustion.

### Procedures

The study was approved by the Canterbury University Human Ethics Committee and conformed to the standards set by the Declaration of Helsinki. Informed voluntary written consent was obtained from each participant prior to the start of the study. Participants were asked to complete two maximal treadmill running tests (using the Bruce and the Athlete-led protocol) to volitional failure in a randomised cross-over trial 1 week apart. The participants were asked to refrain from intense exercise and alcohol for 24 h prior, and caffeine from 4 h prior to each main trial. Participants also recorded their dietary intake prior to the first trial to allow replication of diet prior to subsequent trials. Participants were asked to drink 500 ml of water approximately 2 hours prior to testing in an attempt to standardise bodily fluid concentrations. To assess V̇O_2max_ the participants completed a continuous incremental exercise test to voluntary exhaustion on a calibrated treadmill (Rodby™, RL 1600E, Enhorna, Sweden). Briefly, in the Bruce protocol (Bruce) participants started exercising at a treadmill speed of 2.7 km·h^−1^ and an incline of 10% gradient for 3 minutes. Workloads (speed and inclination) were subsequently increased each 3-minute period in a simultaneous way until volitional exhaustion was reached (Bruce, 1971).

In the athlete-led protocol (ALP) the participants started exercising at an easy running speed (8–10 km·h^−1^) with no incline. This initial speed was set according to the fitness and training status of the individual (8 km·h^−1^ for more sedentary and 10 km·h^−1^ for more athletic participants), thereafter, each subsequent minute the speed was increased by 1 km·h^−1^ until participants reached a pace that was reasonably comfortable and could be maintained for the duration of the test. Once the comfortable running pace was found, the treadmill was increased by a 1% gradient each minute until voluntary exhaustion was reached. Each participant was encouraged to exert a maximal effort. The test was stopped when the participant could not maintain the required pace or had reached voluntary exhaustion. The criterion used to assess V̇O_2max_ included a respiratory exchange ratio ≥ 1.10, a heart rate in excess of 90% of age predicted heart rate maximum (220-age), and identification of a plateau (<150 ml x min^−1^ increase) in V̇O_2max_ despite a further increase in velocity. In all tests, two of the three criteria were met. To enable direct comparison of V̇O_2max_ data between protocols, participants were asked to maintain their normal level of training for the length of the study.

### Measures

Ventilation and expired gases were measured breath-by-breath using a portable gas exchange system (MetaMax® 3B; Cortex Biophysik, Leipzig, Germany). Before testing, the gas analyser was calibrated for volume (Hans Rudolph 5530 3 L syringe; Kansas City, MO, USA) and gas composition (15% O_2_ and 5% CO_2_). Oxygen uptake (V̇O_2_), minute ventilation (V̇_E_), end-tidal CO_2_ (P_ET_CO_2_) and respiratory exchange ratio (RER) were measured. Face masks (Hans Rudolph, Kansas City, MO, USA) with small dead spaces (approximately 70 ml) were fitted to participants allowing simultaneous breathing at the mouth and nose. To de-emphasise breath-to-breath variation, values for V̇O_2_ were smoothed by taking the average for every 15-second time period. During the performance, heart rate was recorded continuously by means of a heart rate monitor (S610; Polar, Kempele, Finland). Blood lactate concentration was determined from a finger-prick sample at rest and 5 minutes post test and analysed using a portable lactate analyser (Lactate Pro, Arkray Inc, Kyoto, Japan). Standing height and nude body mass (Seca scales, Hamburg, Germany) was determined on arrival at the laboratory.

### Analysis

Data was analysed using a specialised spreadsheet for cross-over trials (Hopkins, 2006). We analysed the natural logarithm of each measure to reduce any effects in nonuniformity of error and to obtain changes in measures and errors as percentages. Chances that the true effects were substantial was estimated when a value for the smallest worthwhile effect was entered. We used a value of 5% for V̇O_2max_, because this was considered representative of the smallest worthwhile change for active but non-elite participants. For all other measures we chose 0.20 standardized units (change in mean divided by the between-subject SD at baseline) as the smallest worthwhile change (Cohen, 1988). Uncertainty in the estimate of changes was presented as 90% confidence intervals and as likelihoods that the true value of the effect was a substantial positive or negative difference. Spearman correlations were computed between selected maximal physiological variables collected during each test to provide an indication of overall agreement between the two protocols. We used Cohen’s (Cohen, 1988) guidelines for classifying the correlations (i.e. r < 0.30, small; r = 0.31–0.50, moderate; r > 0.50, large). Bland-Altman plots were used to estimate the agreement between the two test protocols across the range of V̇O_2max_ estimates. The Bland-Altman plot displays the mean of the Bruce and ALP V̇O_2max_ estimates for individuals on the x-axis and the difference between the estimates (Bruce minus ALP) on the y-axis. Correlations between data points on the Bland-Altman plots were used to determine whether there was any form of systematic bias across the range of fitness levels.

## Results

The participants involved in the study are not a representative sample of the total population in terms of fitness levels since the average V̇O_2max_ is substantially higher than what would be normally expected. However, participant’s blood pressure and BMI levels were normal. Most individual’s were involved in some form of recreational sport or activity and trained approximately 4.5 h·wk^−1^ (Table 1).

The ALP protocol produced a mean V̇O_2max_ of 47.02 ± 9.10 ml·kg^−1^·min^−1^ (mean ± SD) compared to the Bruce protocol that produced an overall mean of 46.81 ± 10.7 ml·kg^−1^·min^−1^ (Table 2). There was no substantial difference in V̇O_2max_ between the two protocols calculated in relative or absolute terms. Compared to the Bruce protocol the ALP protocol increased the maximal heart rate during the test (182.8 ± 10.5 and 179.7 ± 8.7 beats·min^−1^, for the ALP and Bruce protocols respectively), however, the 5-minute post-test blood lactate concentrations were similar (ALP = 11.2 ± 3.8 Bruce = 11.7 ± 3.5 mmol·l^−1^). RER was substantially higher in the Bruce compared to the ALP protocol (Table 2). Using the ALP protocol to measure V̇O_2max_ took a substantially shorter period of time (approximately 23 s less) compared to the Bruce protocol. Typical (or standard) error of measurement for the V̇O_2max_ tests was 5.7% (90% CL, 4.6–7.3%).

**Figure 1:**
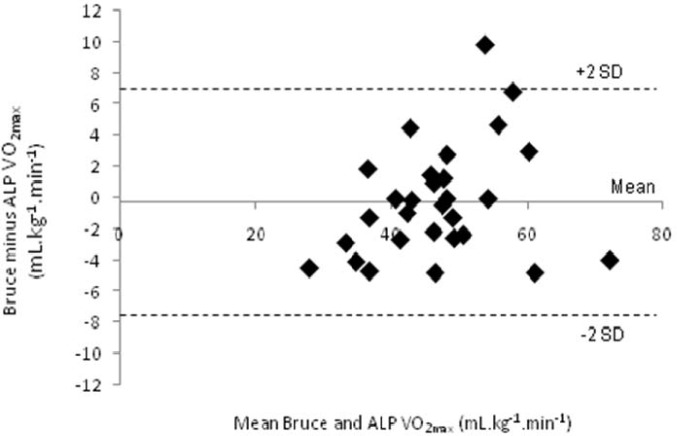
*Agreement between Bruce and ALP protocols for estimating V̇O_2max_*.

In most cases correlations in the physiological variables at maximal exercise between the two protocols were very large (Table 3). Maximal aerobic power (V̇O_2max_) was almost identical whether measured using the Bruce or ALP protocols (r = 0.93). Bland-Altman comparison on the V̇O_2max_ data (Figure 1) indicated good agreement between the two protocols at lower fitness levels, however, variability tended to increase in individuals with higher aerobic power ( > 50 mL·kg^−1^·min^−1^).

Type of protocol (Bruce or ALP) had little effect on relative V̇O_2max_ scores in individuals with different fitness levels (Table 4) and the highest measured V̇O_2max_ tended to be spread evenly among the two protocols.

## Discussion

The major aim of this research was to compare two methods of estimating V̇O_2max_ using progressive treadmill exercise tests to exhaustion. These two tests were highly correlated with V̇O_2max_ being almost identical between the Bruce or ALP protocols and were unaffected by fitness levels of participants, suggesting the two tests are both valid protocols for measuring aerobic capacity.

As suggested by Buchfuhrer and colleagues both treadmill protocols used in this research brought the participants to their tolerance limit within 10 ± 2 min (10:18 and 10:41 min:sec for the ALP and Bruce protocols respectively) (Buchfuhrer et al., 1983), but the Bruce protocol tended to require substantially more time compared to the ALP protocol. This is probably due to the fact that the Bruce protocol starts at a very low intensity (2.7 km·h^−1^ and an incline of 10% gradient) and therefore requires a longer period of time to get participants to their tolerance limit.

Although the V̇O_2max_ was similar between testing protocols the Bruce protocol produced substantially higher RER values compared to the ALP protocol. It is well known that uphill running requires higher energy expenditure than level running (Cavagna et al., 1964). Running up an incline decreases the eccentric component of the muscle contraction thereby reducing the amount of stored energy available in the elastic components of the muscle-tendon complex requiring more energy to come from metabolism (Asmussen and Bonde-Petersen, 1974; Cavagna et al., 1964). It is possible that the Bruce protocol which starts at a more severe incline (10% in Bruce compared to 1% in ALP) may cause participants to recruit their Type II muscle fibres earlier resulting in higher anaerobic metabolism and subsequently higher RER values. Indeed, accumulated oxygen deficit (a marker for anaerobic metabolism) increases with grade of incline in treadmill running (Olesen, 1992) and recent research indicates that a greater amount of lower extremity muscle is activated during uphill running compared to horizontal running (Sloniger et al., 1997). However, an increase in the recruitment of Type II fibres in the Bruce compared to the ALP protocol should increase the resultant blood lactate concentration, but we found no such change. The apparent lack of any substantial change in the blood lactate concentration however, might be explained by the sampling time (blood lactate was sampled 5 min after reaching V̇O_2max_ which may have allowed differences to dissipate).

The substantially higher maximal heart rate obtained when participants completed the ALP compared to the Bruce protocol is an unexpected finding. We suspect the increase in running incline during the Bruce protocol caused increased feelings of fatigue and leg discomfort resulting in participants reaching exhaustion before maximal heart rate was achieved. There is evidence to suggest that V̇O_2max_ test termination may be associated with perceived exertion (Sgherza et al., 2002), rather than any physiological limitation. However this is speculative and requires further research to elucidate the mechanisms behind the maximal heart rate changes witnessed between the protocols used in this study.

## Conclusions

It seems that using the ALP protocol during an incremental treadmill run to exhaustion in a heterogeneous group of individuals is likely to produce similar V̇O_2max_ values compared to the Bruce protocol, irrespective of age, sex or underlying fitness levels. In addition, the ALP protocol also takes a substantially shorter period of time to complete. We therefore suggest that the ALP protocol which is quicker, simpler and better at achieving maximal heart rate values is a useful alternative to the traditional Bruce protocol for testing maximal aerobic fitness.

## Figures and Tables

**Table 1:** Participant Characteristics (n = 29)

Characteristics	Mean ± SD
Age (yr)	29.9 ± 9.7
Body Height (cm)	175.7 ± 8.6
Body Mass (kg)	75.8 ± 10.9
BMI (kg·m^−2^)	24.4 ± 3.1
Resting heart rate (bpm)	59.8 ± 6.9
Resting systolic blood pressure (mm Hg)	125.4 ± 9.8
Resting diastolic blood pressure (mm Hg)	78.8 ± 6.8
Training (h·wk^−1^)	4. ± 2.7

Data are raw means ± SD.

**Table 2:** Physiological and performance values during a maximal running test to exhaustion using the Bruce or ALP protocols, along with the quantitative differences (%), and qualitative inferences. Effects are listed in order of decisiveness.

	ALP mean ± SD	Bruce mean ± SD	% difference; ±90%CL	Qualitative inference
Maximal RER	1.19 ± 0.11	1.24 ± 0.13	4.2;±2.3	Difference likely
Time to V̇O_2max_ (s)	618.0 ± 106.2	641.2 ± 119.8	3.5;±2.6	Difference likely
HR_max_ (beats·min^−1^)	182.8 ± 10.5	179.7 ± 8.7	−1.7;±0.7	Difference likely
Lactate_max_ (mmol·L^−1^)	11.2 ± 3.8	11.7 ± 3.5	4.5;±7.7	Difference trivial
V̇O_2max_ (L·min^−1^)	3.6 ± 0.9	3.6 ± 0.9	−1.0;±2.4	Difference trivial
V̇O_2max_ (ml·kg^−1^·min^−1^)	47.0 ± 9.1	46.8 ± 10.1	−1.0;±2.5	Difference trivial
V̇_E_max__ (L·min^−1^)	132.0 ± 25.7	131.5 ± 26.7	−0.7;±3.4	Difference trivial

*±90CL: add and subtract this number to the mean effect to obtain the 90% confidence limits for the true difference. V̇O_2max_: Maximal aerobic capacity, HR_max_: Maximal heart rate, Lactate_max_: Blood lactate concentration measured 5 min post-test, V̇*_*E*_*max*__*: Maximal ventilation.*

**Table 3:** Agreement between the Bruce and ALP protocols for selected maximal physiological measures.

	V̇O_2max_	Lactate_max_	HR_max_	V̇_E_max__
Total	0.93;±0.05	0.80;±0.12	0.90;±0.06	0.83;±0.10
Age (years)				
18–30 (*n* = 16)	0.94;±0.04	0.65;±0.19	0.86;±0.09	0.87;±0.08
>30 (*n* = 13)	0.86;±0.09	0.82;±0.11	0.76;±0.14	0.76;±0.14
Gender				
Male (*n* = 21)	0.91;±0.06	0.81;±0.11	0.88;±0.08	0.61;±0.20
Female (*n* = 8)	0.97;±0.02	0.80;±0.12	0.90;±0.06	0.62;±0.20

*Data are Spearman correlation coefficients;±0.90% confidence limits. V̇O_2max_: Maximal aerobic capacity, HR_max_: Maximal heart rate, Lactate_max_: Blood lactate concentration measured 5 min post-test, V̇O*_*E*_*max*__*: Maximal ventilation.*

**Table 4:** *V̇O_2max_(ml*·*kg^−1^*·*min^−1^) from the Bruce and ALP protocols for the different fitness groups.*

Fitness Group	Bruce	ALP
Low (*n* = 5)	34.10 ± 5.76	36.13 ± 4.12
Moderate (*n* = 6)	41.15 ± 5.73	42.87 ± 4.74
High (*n* = 18)	52.23 ± 7.73	51.44 ± 8.01

Data are mean ± SD. Fitness levels correspond to age and sex-matched maximal aerobic power from normative tables found in (American College of Sports Medicine, 2006) where low: ≤ 30^th^ percentile, moderate: 31^st^–69^th^ percentile, high: ≥ 70^th^ percentile.
